# General dimensions of human brain morphometry inferred from genome‐wide association data

**DOI:** 10.1002/hbm.26283

**Published:** 2023-03-29

**Authors:** Anna E. Fürtjes, Ryan Arathimos, Jonathan R. I. Coleman, James H. Cole, Simon R. Cox, Ian J. Deary, Javier de la Fuente, James W. Madole, Elliot M. Tucker‐Drob, Stuart J. Ritchie

**Affiliations:** ^1^ Social, Genetic and Developmental Psychiatry (SGDP) Centre Institute of Psychiatry, Psychology & Neuroscience, King's College London London SE5 8AF UK; ^2^ National Institutes for Health Research Maudsley Biomedical Research Centre South London and Maudsley NHS Trust London SE5 8AF UK; ^3^ Department of Neuroimageing Institute of Psychiatry, Psychology & Neuroscience, King's College London London SE5 8AF UK; ^4^ Centre for Medical Image Computing, Department of Computer Science University College London London WC1V 6LJ UK; ^5^ Dementia Research Centre Institute of Neurology, University College London London WC1N 3BG UK; ^6^ Department of Psychology The University of Edinburgh Edinburgh EH8 9JZ UK; ^7^ Lothian Birth Cohorts University of Edinburgh Edinburgh EH8 9JZ UK; ^8^ Department of Psychology University of Texas at Austin Austin Texas 78712‐1043 USA; ^9^ Population Research Center and Center on Ageing and Population Sciences University of Texas at Austin Austin Texas 78712‐1043 USA

**Keywords:** brain age, cognitive ability, complex traits, genetics, statistical modelling, structural brain networks, structural neuroimageing

## Abstract

Understanding the neurodegenerative mechanisms underlying cognitive decline in the general population may facilitate early detection of adverse health outcomes in late life. This study investigates genetic links between brain morphometry, ageing and cognitive ability. We develop Genomic Principal Components Analysis (*Genomic PCA*) to model general dimensions of brain‐wide morphometry at the level of their underlying genetic architecture. Genomic PCA is applied to genome‐wide association data for 83 brain‐wide volumes (36,778 UK Biobank participants) and we extract genomic principal components (PCs) to capture global dimensions of genetic covariance across brain regions (unlike ancestral PCs that index genetic similarity between participants). Using linkage disequilibrium score regression, we estimate genetic overlap between those general brain dimensions and cognitive ageing. The first genetic PCs underlying the morphometric organisation of 83 brain‐wide regions accounted for substantial genetic variance (*R* ^*2*^ = 40%) with the pattern of component loadings corresponding closely to those obtained from phenotypic analyses. Genetically more central regions to overall brain structure ‐ specifically frontal and parietal volumes thought to be part of the central executive network ‐ tended to be somewhat more susceptible towards age (*r* = −0.27). We demonstrate the moderate genetic overlap between the first PC underlying each of several structural brain networks and general cognitive ability (*r*_*g*_ = 0.17–0.21), which was not specific to a particular subset of the canonical networks examined. We provide a multivariate framework integrating covariance across multiple brain regions and the genome, revealing moderate shared genetic etiology between brain‐wide morphometry and cognitive ageing.

## Graphical abstract

Understanding the neurodegenerative mechanisms underlying cognitive declines in the general population may facilitate early detection of adverse health outcomes in late life. By modelling structural brain networks on the basis of their underlying genetic architecture, we explore genetic links between brain organisation, cognitive ability and ageing and demonstrate substantial shared genetic etiology between brain organisation and factors that all have potential social and economic consequences for ageing societies.

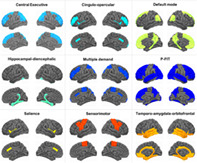

## 1 INTRODUCTION

Progressive ageing‐related neurodegenerative processes in the human brain are well‐documented across the micro‐ and macro‐scales within otherwise healthy adults and are linked to ageing‐related declines in multiple domains of cognitive function (Cox et al., 2016; Fjell & Walhovd, 2010; Madole et al., 2021). Understanding the biological processes underlying these links is paramount for identifying mechanisms of cognitive ageing that can ultimately be targeted by the intervention. The human brain is a complex network of partially functionally and anatomically overlapping and interconnected regions (Bressler & Menon, 2010; Power et al., 2011; Sporns, 2011; Yeo et al., 2011), whose components age unevenly over time (Raz et al., 2010), and may be differentially relevant to adult cognitive ageing (Cox et al., 2019; Fjell & Walhovd, 2010; Madole et al., 2021).

Whereas considerable attention has been devoted separately to the genetic architecture of human brain morphometry (Anderson et al., 2021; van der Meer et al., 2021; Zhao, Luo, et al., 2019) and the genetic architecture of adult cognitive ability (de la Fuente et al., 2021), relatively less work has explicitly linked investigations of the genetic architecture of human brain morphometry to the putative organisation of brain networks (although see [Arnatkevičiūtė et al., 2021] for a recent exception). In addition, there have been few investigations of how genetic links between components of human brain networks relate to ageing and cognition.

**FIGURE 1:**
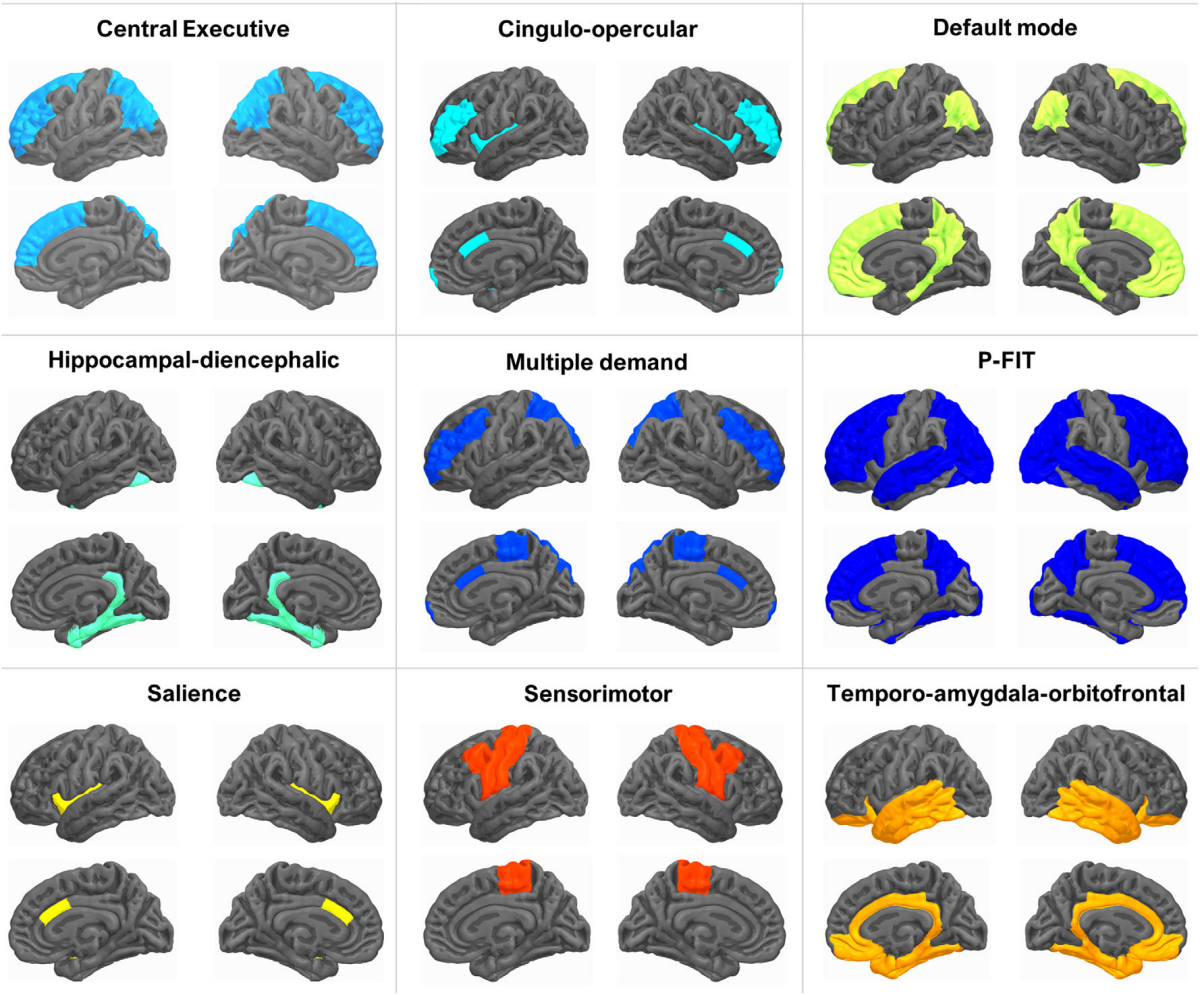
Canonical brain network definitions. To scaffold the genetic architecture of human brain morphometry onto the canonical network organisation of the brain, we consider nine overlapping brain networks. Regional volumes thought to reside within these networks are represented through genome‐wide association data of 83 grey‐matter volumes (*N* = 36,778), and this Figure indicates which networks different volumes were allocated to. The network definitions were adopted from Madole et al. (2021), but are not indisputable. We used these theory‐based network definitions to apply our novel dimensionality reduction technique *Genomic PCA*, to obtain genetic PCs underlying clearly labelled networks. Using these genetic PC1s, we tested whether different networks, or even the whole brain are genetically associated with cognitive ageing.

To model the underlying genetic architecture of brain organisation, we developed Genomic Principal Component Analysis (*Genomic PCA*), a multivariate approach in which we integrate multiple regional brain volumes and the genome to model general dimensions of brain structure. Using genome‐wide association study (GWAS) summary statistics as input, Genomic PCA extracts genetic principal components (PCs) underlying multiple GWAS phenotypes (unlike the ancestry‐based PCs commonly used in genomic research that index genetic similarity between participants). Genetic PCs underlying the whole brain, as well as nine groups of regional brain volumes that reflect canonical brain networks (Figure 1) are then tested for associations with cognitive ability and ageing. This genetically‐informed approach parallels a previous study modelling *phenotypic* PCs underlying the same canonical brain networks, which showed that frontal and parietal brain volumes—part of the central executive network—were more important to overall brain structure (i.e., higher loadings onto a PC underlying the whole brain), and tended to have stronger cross‐sectional associations with age than other regions of the brain (*N* = 8185) (Madole et al., 2021).

The canonical brain networks examined here are based on a whole‐brain perspective, considering the existing literature that describes synchronised (i.e., correlated) regional activity in functional magnetic resonance imaging (MRI) data (Madole et al., 2021), in addition to converging evidence from other modalities (i.e., structural MRI and lesion‐based mapping) (Bressler & Menon, 2010; Jung & Haier, 2007; Menon & Uddin, 2010). Among the most reported networks are the central executive (Sridharan et al., 2008), default mode (Buckner & DiNicola, 2019), salience (Downar et al., 2002) and multiple demand networks (Duncan, 2010). Our investigation focuses on brain volumes within these networks because they are highly heritable (Zhao, Ibrahim, et al., 2019) and are measured independently of mental processes during MRI scanning (compared with functional MRI). Grey matter volume is a robust predictor of general cognitive ability (Cox et al., 2019; Hilger et al., 2020), and it partly reflects age‐related atrophy among middle‐and‐older adults; an important indicator of ageing and health outcomes (Cole et al., 2018).

There are substantial genetic links between brain structure and cognitive function in ageing. For example, a recent investigation ran a GWAS on the *brain age* gap, which is an index of how much older (or younger) an individual's brain appears compared to their chronological age. Substantial genetic correlations were revealed between a dementia screening test (Mini‐Mental State Examination) and brain age in the whole brain (*r*_*g*_ = −0.3), as well as the four brain lobes (*r*_*g*_ = −0.15 to −0.22), suggesting that there is a genetic component to how quickly one's brain degrades with age.

Overall brain volume and cognitive ability are also genetically correlated (*r*_*g*_ = 0.24), implicating genes involved in regulating cell growth (Jansen et al., 2020). Biton et al. (2020) reported smaller genetic correlations between intelligence and seven regional brain volumes (range *r*_*g*_ = 0.07–0.13), which is the only study we are aware of that considered regional volumes *not* normalised for global brain measures. Studies normalising for global measures report only small, or even negative associations between cognitive ability and regional brain structures (e.g., *r*_*g*_ = −0.13 between intelligence and frontal lobe de Vlaming et al., 2021; see also Grasby et al., 2020; Zhao, Luo, et al., 2019), which is of secondary interest to our study because this only considers regional variance above and beyond variance that maps onto total brain size. Instead, we consider regional variance central to overall brain structure: rather than discarding it (and the regional information it carries; Reardon et al., 2018), we model interregional variance because cognitive ability and ageing are brain‐wide distributed phenomena (Cole et al., 2019; Hilger et al., 2020), that are more associated with brain features shared between regions (rather than noisy region‐specific brain features) (Cox et al., 2021).

The aims of this pre‐registered study are twofold (https://osf.io/7n4qj). First, we link investigations of the genetic architecture of human brain morphometry with canonical brain networks, to test whether genetics operate on the same dimensions as are evident phenotypically. As Cheverud originally speculated, “If genetically and environmentally based phenotypic variations are produced by similar disruptions of developmental pathways, genetic and environmental correlations should be similar” (Cheverud, 1988). We therefore hypothesised a close correspondence of phenotypic and genetic morphometric correlations (as demonstrated across a range of traits in Biton et al., 2020; Sodini et al., 2018). A dissimilar organisation of phenotypic and genetic brain architecture would raise questions regarding the neurobiological validity of canonical brain networks in interindividual differences of structural grey matter. A similar organisation would be consistent with a measurable genetic foundation of structural brain networks.

Second, we investigate the extent to which genetic correlations among brain organisation, cognitive ability and ageing corroborate the magnitude and direction of well‐established phenotypic associations. We hypothesised substantial genetic correlations of these variables with general morphometric dimensions across the whole brain, and nine overlapping structural brain networks. As implied by the phenotypic results of Madole et al. (2021), we expected the central executive network to play a disproportionate role in cognitive ability, which would confirm a more precise neurobiological foundation of cognitive ability.

## 2 METHODS

**FIGURE 2:**
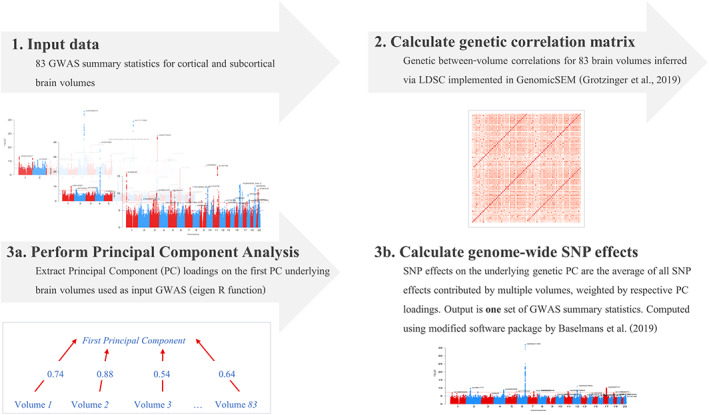
Genomic PCA pipeline. (1) Input data: The pipeline takes GWAS summary statistics as input. Here, we calculated GWAS summary statistics for 83 cortical and subcortical grey‐matter volumes, which were the input to the analyses presented throughout the manuscript. (2) Calculate genetic correlation matrix: We calculated interregional genetic correlations based on LDSC as implemented in GenomicSEM (Grotzinger et al., 2019). (3a) Perform PC Analysis: We performed Eigendecomposition of the genetic correlation matrix using the Eigenfunction in R in order to extract PC1 loadings on the first PC underlying brain volumes for which we submitted GWAS summary statistics to the pipeline. Here we obtained PC1 loadings for each of the 83 brain volumes, and an estimate of *R* ^*2*^ quantifying how much genetic variance PC1 explained across all input volumes. (3b) Calculate genome‐wide SNP effects: To obtain genome‐wide SNP‐wise effects on the underlying genetic PC1, we calculated each SNP effect as the average of all SNP effects contributed by the input volumes, weighted by respective volume‐specific PC1 loadings. This created one set of GWAS summary statistics representative of genetic correlates of an underlying genetic PC1. Individual SNP effects were computed with a modified function by Baselmans et al. (2019). We used the same procedure to also obtain PC1s underlying different brain networks, for which we submitted fewer volumes as input.

The UK Biobank sample consisted of 36,778 unrelated White European participants (54% females) with available neuroimageing data. They had an average age of 63.3 years at neuroimageing visits (range from 40.0 to 81.8 years; Supplementary Methods 1.1). Standard quality checks were performed as described in Supplementary Methods 1.2–1.3. We derived *Genomic Principal Components Analysis* (G*enomic PCA*; Figure 2) that follows three major steps to extract general dimensions of human brain morphometry underlying genetic covariance across multiple brain GWAS phenotypes (unlike ancestral PCs that index genetic similarity between participants).

**FIGURE 3:**
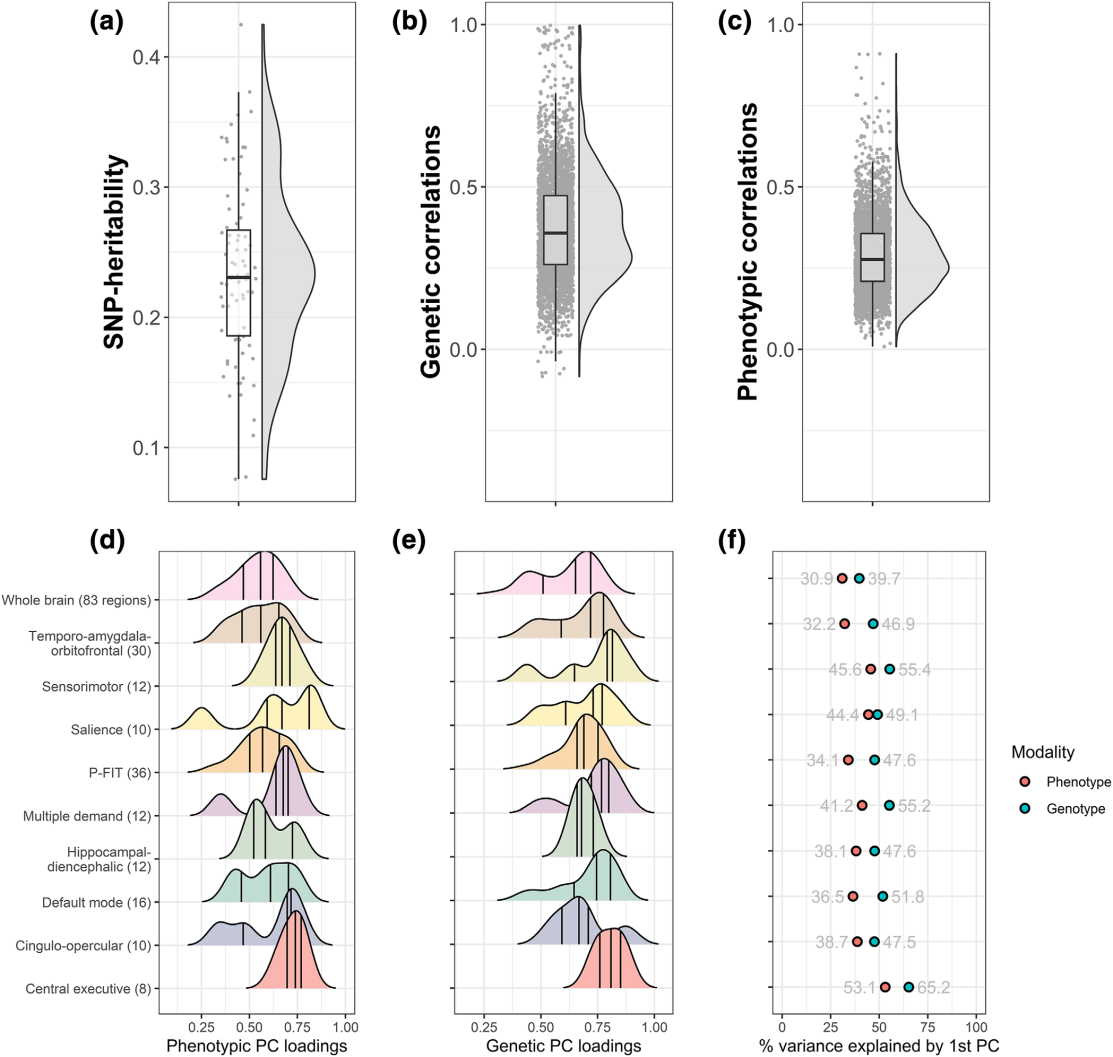
Descriptive statistics. (a) Distribution of SNP‐heritability estimates for 83 regional grey‐matter volumes inferred through univariate LDSC. (b) Distribution of genetic correlations among 83 regional grey‐matter volumes inferred through between‐volume LDSC. (c) Distribution of phenotypic correlations among 83 regional grey‐matter volumes inferred through Pearson's correlations. Raincloud plots were created based on code adapted from Allen et al. (2019). *Bottom row*: Density distributions of PC1 loadings on the first PC underlying volumes in (d) phenotypic and (e) genetic networks. Vertical lines indicate quantiles. Genetic PC1 loadings are plotted onto corresponding brain regions in Supplementary Figure 24. (f) Variance explained by phenotypic and genetic first PC1 underlying volumes in each network.

First, we calculated 83 GWAS summary statistics for 83 cortical and subcortical grey‐matter volumes (33 cortical Desikan‐Killiany [Desikan et al., 2006] regions in each hemisphere +8 subcortical regions in each hemisphere + brain stem; Figure 2.1). UKB field IDs are listed in Supplementary Table 1. GWAS effects were fitted in a linear mixed model using REGENIE (Mbatchou et al., 2021). SNP‐heritability for each volume was comparable to those reported elsewhere (Zhao, Ibrahim, et al., 2019) (mean = 0.23, range = 0.07–0.42; Figure 3a).

Second, we calculated genetic correlation matrices indicating genetic overlap between the 83 volumes using linkage disequilibrium score regression (LDSC; Bulik‐Sullivan et al., 2015) as implemented in the GenomicSEM software (Grotzinger et al., 2019; Figure 2.2). Genetic between‐volume correlations are displayed in Supplementary Figures 1–10.

Third, we extracted the first genetic principal component (PC1) underlying genetic variance shared across multiple GWAS phenotypes (here we used 83 brain volumes as input), by which we reduced dimensionality from multiple to only one set of GWAS summary statistics. PC1 loadings and *R* ^*2*^ estimates were calculated with the Eigenfunction in R (Figure 2.3a). Genome‐wide SNP effects are calculated as the average of SNP effects from multiple GWAS phenotypes weighted by (volume‐specific) PC1 loadings. Standard errors are corrected for sample overlap by taking into account LDSC intercepts (Figure 2.3b). In cases of complex and highly dimensional data (e.g., large numbers of variables, or complex loading structure making a factor model in GenomicSEM [Grotzinger et al., 2019] unfeasible), Genomic PCA permits a focus on the first dimension of maximal variation without assuming that there is only one dimension (which is what fitting a one‐factor model would require). Genomic PCA is also computationally simpler given a large number of considered ROIs. It is a major advantage that no access to individual‐level phenotype data is needed to perform Genomic PCA, and we validated the approach by demonstrating that GWAS summary statistics produced by Genomic PCA are very similar (*r*_*g*_ = 0.99) to GWAS summary statistics obtained from running GWAS analyses on a phenotypic PC1 (more details at https://annafurtjes.github.io/genomicPCA/, and in Supplementary Methods 2.5).

Using Genomic PCA, we performed theory‐driven dimensionality reduction by extracting genetic PC1s from covariance structures across nine canonical brain networks (as well as the whole brain with 83 regions). That is, we submitted groups of brain volumes to Genomic PCA that are thought to be part of canonical brain networks (Supplementary Table 2 lists volumes allocated to nine overlapping networks). Network definitions have been adopted from Madole et al. (2021), where networks were aligned with the structural, functional and lesion‐based literature (e.g., Bressler & Menon, 2010; Jung & Haier, 2007; Menon & Uddin, 2010).

The remainder of the Methods outlines analyses of genetic PC1s underlying multiple brain volumes derived with Genomic PCA and is structured according to the four major sub‐sections of the results:

1. First, we reported summary statistics (including volumetric PC1 loadings and variance explained by PC1; *R* ^*2*^) describing the genetic PC1s underlying the whole brain (83 regions), as well as nine canonical brain networks including fewer regions (Section 3.1).
2. Second, we tested whether genetic interregional covariance is similarly organised to phenotypic interregional covariance. To obtain comparable indices of phenotypic covariance, we ran a standard (phenotypic) PCA on a phenotypic correlation matrix obtained from the same brain volume variables used to calculate GWAS. Phenotypic PCA was performed with the Eigenfunction in R, which is also used in Genomic PCA. We quantified linear associations and the Tucker congruence coefficient (Lorenzo‐Seva & Berge, 2006) to contrast genetic and phenotypic interregional correlations, as well as genetic and phenotypic PC1 loadings underlying brain‐wide volumes (Section 3.2).
3. Third, to quantify the genetic relationship between general dimensions of brain morphometry and cognitive ability, we extracted a general factor of cognitive ability in GenomicSEM (Grotzinger et al., 2019) using factor analysis of seven cognitive traits as published by de la Fuente et al. (2021). The seven cognitive traits were *Matrix Pattern Completion task* for nonverbal reasoning, *Memory*—*Pairs Matching Test* for memory, *Reaction Time* for perceptual motor speed, *Symbol Digit Substitution Task* for information processing speed, *Trail Making Test*—*B* and *Tower Rearranging Task* for executive functioning and *Verbal Numerical Reasoning Test* for verbal and numeric problem solving, or fluid intelligence. The main results of this section are genetic correlations between general cognitive ability and genetic PC1s underlying the whole brain and nine different brain networks (Section 3.3). Additionally, we report genetic correlations with individual cognitive abilities, *Q*_*trait*_ analyses (Grotzinger et al., 2020) and we test whether the central executive network is particularly relevant for cognitive ability (Supplementary Methods 2.11).
4. Fourth, we tested for associations between general dimensions underlying the whole brain and age‐related indices to understand whether generally more important regions for overall brain structure are also more susceptible to cognitive ageing (which would support shared mechanisms). This fourth section is split into two parts: First, we tested for a linear association between the genetic PC1 loadings of all 83 volumes (onto a PC1 underlying the whole brain) and a volume's cross‐sectional association with age (Section 3.4.1), which has previously been called its “age sensitivity” (Madole et al., 2021). This analysis was not repeated for smaller subnetworks, because the low degree of statistical power did not allow us to meaningfully estimate the correlation between the vectors.

In a second, non‐registered analysis, we quantified a genetic correlation between a genetic PC1 underlying the whole brain and the brain age gap (the gap between chronological and biological brain age), for which we utilised GWAS summary statistics by Kaufmann et al. (2019). This brain age gap GWAS was based on the difference between an individual's chronological age and age predictions of how old (or young) an individual's brain appears from structural MRI measures (Section 3.4.2). This analysis was only performed for a genetic PC1 underlying the whole brain, but not PC1s underlying different networks, because the different PC1s were so strongly associated that they indexed practically the same polygenic signal (as discussed in the last paragraph Section 3.1). More details on Methods are in Supplementary Methods. Our analysis code is displayed at https://annafurtjes.github.io/Genetic_networks_project/.

## 3 RESULTS

### 3.1 Descriptive statistics of genomic PC1s underlying whole brain and canonical brain networks

#### 3.1.1 Genetic PC1s underlying volumes across the whole brain

In this section, we report variance explained (*R* ^*2*^) by the first underlying volumetric PC (PC1) and corresponding PC1 loadings obtained from Genomic PCA of the whole brain (83 regions), as well as nine overlapping canonical brain networks. The PC1 underlying the whole brain explained 40% of the genetic variance across 83 regional volumes—slightly larger than the 31% explained by the first phenotypic whole‐brain PC1 (Figure 3f). For comparison, the second genetic PC2 accounted for a fraction of the variance that the first PC1 explained (*R* ^*2*^ = 6.7%), indicating that the first genetic PC1 accounted for the majority of systematic variance across structural networks. Genetic PC1 loadings onto the first PC1 underlying the whole brain ranged between 0.30 and 0.81 (mean = 0.62, *SD* = 0.13, median = 0.65; Figure 3e, Supplementary Table 3).

#### 3.1.2 Genetic PC1s underlying volumes in canonical networks

The first genetic PC1s underlying different brain networks accounted for greater *R* ^*2*^ than the genetic whole‐brain PC1. *R* ^*2*^ ranged from 65% explained by the first genetic PC1 underlying the central executive network, to 47% accounted for by the first genetic PC1 underlying the temporo‐amygdala‐orbitofrontal network (Figure 3e). *R* ^*2*^ was larger for networks including fewer volumes, which tended to be more homogeneous, as indicated by PC1 loadings (e.g., range 0.74–0.88 for central executive, range 0.43–0.89 for sensorimotor). Parallel Analysis confirmed that genetic PC1s underlying all brain networks explained substantially more variance than expected by chance (Scree Plots Supplementary Figures 11–20). Further simulations demonstrated that our theoretical grouping of volumes into networks resulted in more variance explained than expected by randomly grouping volumes (Supplementary Table 5; Supplementary Methods 2.7).

To compare the polygenic signal captured by different brain networks, we calculated genetic correlations between them using Linkage Disequilibrium Score Regression (LDSC; Bulik‐Sullivan et al., 2015). Those genetic correlations tended to be very high (mean *r*_*g*_ between networks 0.83, SD = 0.09; range = 0.63–0.97), suggesting different network PC1s captured roughly the same polygenic signal. For example, the central executive network was genetically associated with the whole brain at *r*_*g*_ = 0.91. That is, we obtained practically the same polygenic signal when extracting a genetic PC1 from the whole brain (83 volumes), as we obtained from extracting a genetic PC1 from fewer volumes (e.g., 8 volumes in the central executive).

### 3.2 Comparing genetic and phenotypic interregional covariance

**FIGURE 4:**
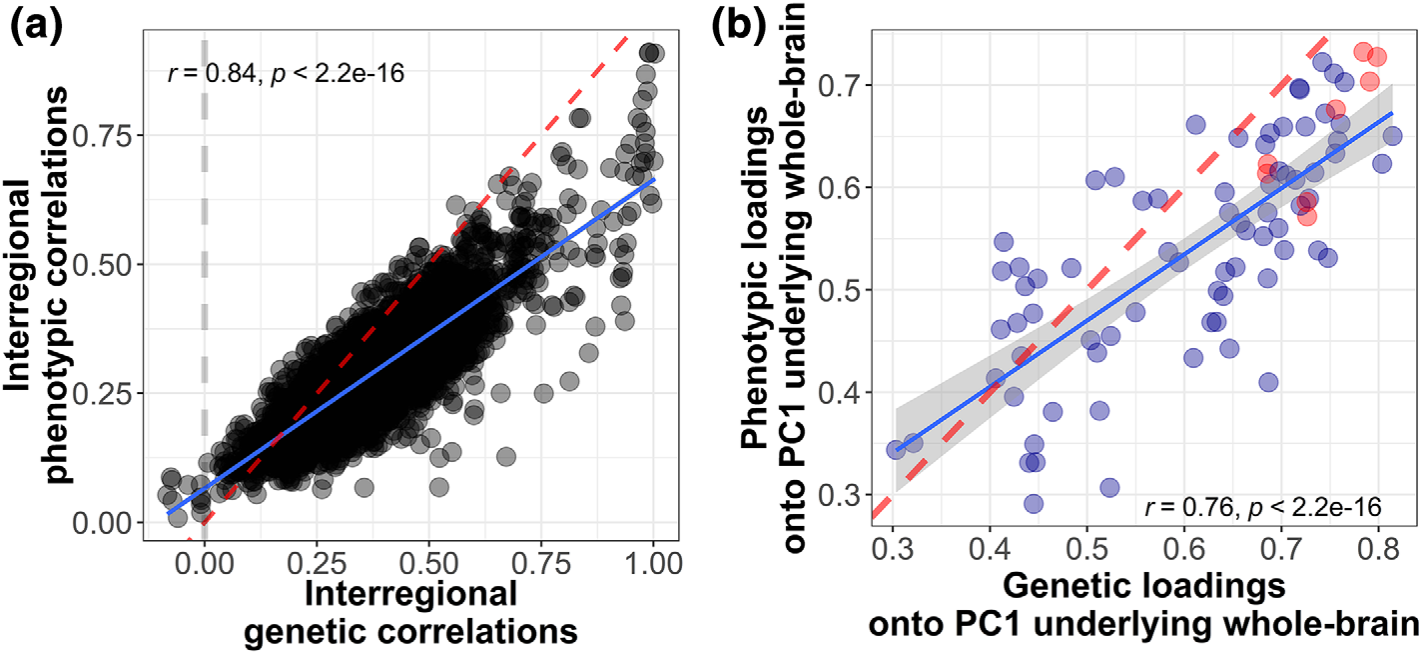
Quantitative comparison of phenotypic and genetic interregional covariance. Figure (a) is contrasting 4303 between‐volume correlations where the phenotypic correlations were obtained from phenotypic brain volumes, and the genetic correlations were obtained from LDSC of GWAS summary statistics of the same brain volumes. Figure (b) contrasts 83 phenotypic and genetic PC1 loadings onto an underlying whole‐brain PC1. Regions coloured in red are regions allocated to the central executive network, which tend to be both phenotypically and genetically central to overall brain structure (i.e., high PC1 loadings).

**FIGURE 5:**
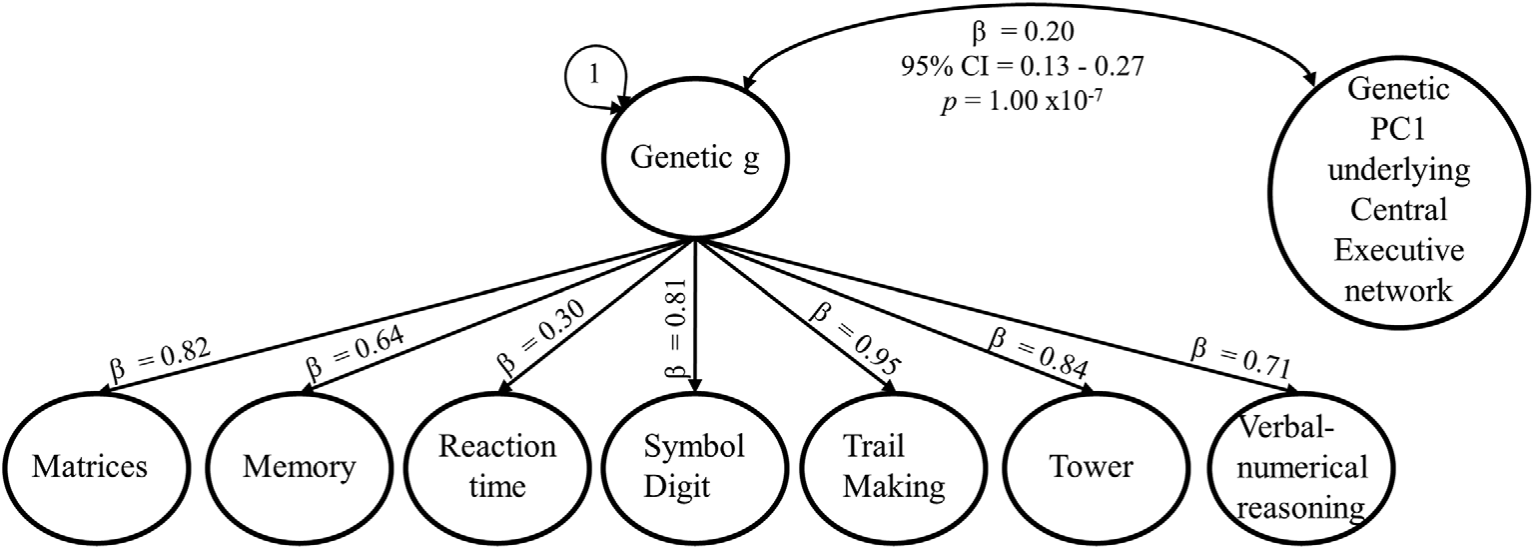
Genomic Structural Equation Model calculating genetic correlations between general cognitive ability and genetic PC1s. We modelled a genetic *g‐*factor of general cognitive ability in GenomicSEM (Grotzinger et al., 2019) using cognitive ability GWAS summary statistics obtained from de la Fuente et al. (2021). The genetic correlation between genetic *g* and general morphometric dimensions underlying the whole brain and nine canonical brain networks (modelled using Genomic PCA) are reported in Table 1. The seven cognitive traits and the networks are inferred through LDSC. Matrix, matrix pattern completion task; Memory, memory—pairs matching test; RT, reaction time; Symbol Digit, symbol digit substitution task; Trails‐B, trail making test – B; Tower, tower rearranging task; VNR, verbal numerical reasoning test. Model fit: *χ* ^*2*^ = 124.04, df = 20, *p*‐value = 2.1 × 10^−20^, AIC = 174.04, CFI = 0.97, SRMR = 0.079.

To quantify how indices of genetic and phenotypic interregional covariance resemble each other, we calculated linear associations between phenotypic and genetic between‐volume correlations, as well as linear associations and Tucker congruence coefficient between phenotypic and genetic PC1 loadings onto an underlying whole‐brain PC1. The vectors of 3403 phenotypic and 3403 genetic interregional correlations were strongly positively associated (*r* = 0.84; *b* = 0.60; SE = 0.007, *p* < 2 × 10^−16^, *R* ^*2*^ = 70%) (Figure 4a), indicating that volumes that were strongly phenotypically correlated were also strongly genetically correlated. Magnitudes of genetic correlations tended to be slightly larger than phenotypic correlations (intercept = 0.06) which is consistent with previous reports (Biton et al., 2020) (Figure 5a).

The association between phenotypic PC1 loadings and genetic PC1 loadings was large and significant (*b* = 0.65, SE = 0.06, *p* = 5.07 × 10^−17^, *R* ^*2*^ = 58%, intercept = 0.15; Figure 4b). The Tucker congruence coefficient was used to index the degree of similarity between genetic and phenotypic PC1 loadings, taking into account both their relative ordering and their absolute magnitudes (Lorenzo‐Seva & Berge, 2006). It revealed very high congruence between phenotypic and genetic PC1 loadings for the 83 volumes (Tucker coefficient = 0.99). These results illustrate a close correspondence and an equivalent organisation of phenotypic and genetic dimensions of shared morphometry; a finding that aligns with Cheverud's Conjecture (Section 4.2).

### 3.3 Genetic correlations between general cognitive ability and general dimensions of human brain morphometry

To quantify the genetic relationship between general dimensions underlying brain morphometry with cognitive ability, we fitted a general factor of cognitive ability (genetic *g*) indicated by seven cognitive test GWAS in GenomicSEM (Grotzinger et al., 2019) and calculated its genetic correlation with genetic PC1s underlying brain volumes in different brain networks (Figure 5). The whole brain and all network‐specific genetic PC1s were significantly genetically associated with general cognitive ability. Correlation magnitudes ranged between *r*_*g*_ = 0.17–0.21 (Table 1). According to commonly‐used rules of thumb from Hu and Bentler (1998) (CFI >0.95, RMSEA <0.08), all models showed good model fit (Supplementary Table 4).

**TABLE 1:** Genetic correlations (*r*_*g*_) between general cognitive ability and general dimensions of morphometry underlying the whole brain and nine canonical brain networks.

Network	Included volumes	*r*_*g*_	95% CI	*p*‐value	FDR *q*‐value
Whole brain	83	0.21	0.13–0.29	1.00 × 10^−7^	3.00 × 10^−7^
Central executive	8	0.20	0.12–0.27	1.00 × 10^−7^	3.00 × 10^−7^
Cingulo‐opercular	10	0.20	0.13–0.27	1.00 × 10^−7^	3.00 × 10^−7^
Default mode	16	0.19	0.12–0.26	2.00 × 10^−7^	3.00 × 10^−7^
Hippocampal‐diencephalic	12	0.17	0.09–0.24	2.66 × 10^−5^	2.66 × 10^−5^
Multiple demand	12	0.19	0.12–0.27	7.00 × 10^−7^	9.00 × 10^−7^
P‐FIT	36	0.20	0.12–0.27	2.00 × 10^−7^	3.00 × 10^−7^
Salience	10	0.19	0.12–0.26	3.00 × 10^−7^	4.00 × 10^−7^
Sensorimotor	12	0.19	0.11–0.27	1.20 × 10^−7^	1.30 × 10^−6^
Temporo‐amygdala‐orbitofrontal	30	0.20	0.12–0.27	2.00 × 10^−7^	4.00 × 10^−7^

Abbreviations: 95% CI, 95% confidence interval; FDR, false discovery rate; *p*‐value, original *p*‐value as indicated by the GenomicSEM model; *q*‐value, *p*‐value corrected using 5% false discovery rate; *r*_*g*_, genetic correlation between genetic PC1s underlying nine canonical brain networks and a factor of general cognitive ability modelled from seven cognitive traits; SE, standard error.

We also report genetic correlations for three individual cognitive traits, because the available GWAS data (de la Fuente et al., 2021) did not warrant modelling separate cognitive domains. Each domain had a maximum of two traits only (e.g., logical reasoning is assessed by both Matrix Pattern Completion and Verbal Numerical Reasoning). Some cognitive tests were impure and contained various cognitive components (e.g., the Trail Making Test assesses executive and speed abilities). To reduce the multiple testing burden, we pre‐registered (https://osf.io/7n4qj) genetic correlations for three cognitive tests that assess relatively separate cognitive abilities: *Matrix Pattern Completion* consistently yielded the strongest genetic correlations with PCs underlying the brain networks (mean *r*_*g*_ across different networks = 0.18). Genetic correlations for *Symbol Digit Substitution Task* were slightly smaller (mean *r*_*g*_ = 0.12), followed by *Memory* which had the lowest average correlations (mean *r*_*g*_ = 0.09).

The significant genetic correlations—between general cognitive ability and genetic PC1s underlying different brain networks—seem to act through a factor of general cognitive ability, rather than through individual cognitive abilities, because individual cognitive traits had high loadings on the genetic cognitive ability factor (median = 0.81, range = 0.30–0.95; Supplementary Figure 22). Also, *Q*_*trait*_ heterogeneity analyses (Grotzinger et al., 2020) demonstrated that the general cognitive ability factor accounted well for the patterns of association between specific cognitive abilities and brain network genetic PC1s (Supplementary Figure 23). That is, models allowing independent associations for all individual cognitive traits did not yield better model fit than models forcing any association to go through the general cognitive ability factor (∆ *χ* ^*2*^ *~* 0; *df* = 6; Supplementary Table 5).

Based on previous phenotypic findings that highlighted the importance of the central executive network to general cognitive ability (Madole et al., 2021), we hypothesised to finding stronger genetic correlations between general cognitive ability and volumetric PC1s underlying the central executive network, relative to other brain networks (see pre‐registered plan https://osf.io/7n4qj). In our genetic analyses, there was no evidence for differences in correlation magnitudes between the central executive network and general cognitive ability compared with other networks, even after accounting for network sizes (Supplementary Figure 22; Supplementary Table 6). Adjustments for network sizes were done by dividing effect sizes by the number of volumes contained in a network (Supplementary Methods 2.11).

### 3.4 Associations between ageing and general dimensions of brain morphometry

#### 3.4.1 Associations between genetic whole‐brain PC1 loadings and age sensitivity

**FIGURE 6:**
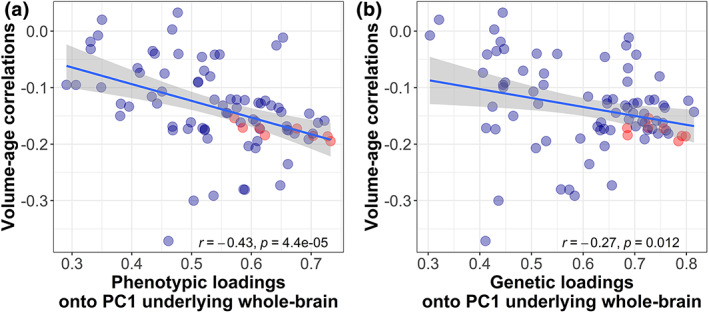
Association between (a) phenotypic and (b) genetic PC1 loadings of all 83 volumes (onto a PC1 underlying the whole brain) and a volumes' cross‐sectional association with age (Section 3.4.1), which is known as “age sensitivity” (Madole et al., 2021). Volumes coloured in red are regions thought to reside in the central executive network, which tended to be both phenotypically and genetically central to overall brain structure (high PC1 loadings), and they tended to be more susceptible towards age (large volume‐age correlation).

Previous phenotypic work demonstrated that brain volumes more central to overall brain structure—indexed by PC1 loadings onto a phenotypic PC1 underlying 83 brain‐wide volumes—were most susceptible to ageing. Ageing was represented by cross‐sectional Pearson's volume‐age correlations (Madole et al., 2021), which are typically negative in adult populations. Here, we replicated this phenotypic association in a larger sample (*r* = −0.43, *p* = 4.4 × 10^−5^; Figure 6a), and we found a significant, though smaller association between *genetic* PC1 loadings and the same volume‐age correlations (*r* = −0.27, *p* = 0.012; Figure 6b). This suggests that the more genetically central a region was to the overall brain structure, the more sensitive that region also was to age‐related shrinkage. Note that this association with age sensitivity emerged even though the PC1 loadings were extracted from brain volume GWAS residualised for age.

#### 3.4.2 Genetic correlation between whole‐brain genetic PC1 and brain age gap

Finally, we calculated a genetic correlation between a genetic PC1 underlying the whole brain and *brain age*, for which we used the brain age GWAS by Kaufmann et al. (2019). The genetic correlation was moderate and negative (*r*_*g*_ = −0.34; *SE* = 0.06), suggesting that there is a shared genetic basis for demonstrating younger brain age, and having consistently larger volumes across the whole brain (ageing discussion in Section 4.3).

## 4 DISCUSSION

Here, we have introduced a multivariate approach integrating covariance across both multiple brain regions and the genome (*Genomic PCA*) to help understand the links between the genetic architecture of human brain morphometry and the network organisation of the brain. In line with Cheverud's Conjecture (Cheverud, 1988), phenotypic and genetic brain organisation seemed to operate on the same major dimensions: phenotypic and genetic correlations were similar (Section 4.1). There was moderate genetic overlap between cognitive ability, ageing and global trends of morphometry underlying both the whole brain and more parsimonious canonical brain networks (Section 4.2 for cognitive ability, Section 4.3 for ageing). To complement theory‐driven perspectives like in this study, our method Genomic PCA may be used to identify regions most important to overall brain structure (e.g., volumes with the largest PC loadings) to be prioritised in future investigations of the relationship between the brain and cognitive ability.

### 4.1 Analogous phenotypic and genetic interregional covariance across the brain

To our knowledge, this is the first genetically‐informed study that corroborates the brain organisation observed in phenotypic studies ‐ we demonstrated analogous interregional covariance across the whole brain derived from both phenotypic and genetic indices (i.e., highly corresponding interregional correlations and whole‐brain PC1 loadings). Analogous to phenotypic findings in Madole et al. (2021), we found that some volumes were genetically more important for overall brain structure than others, indicated by high loadings onto the first PC underlying the whole brain. For example, frontal and parietal volumes theorised to be part of the central executive network, had consistently high loadings, indicating their overall importance for overall brain structure.

The close phenotypic and genetic correspondence in interregional covariance means that inferences from genetic to phenotypic dimensions are viable. This is in line with previous studies comparing phenotypic and genetic correlations between morphometric traits (Biton et al., 2020; Sodini et al., 2018). According to Cheverud's Conjecture, this indicates that genetics of brain organisation operate on the same dimensions as are evident phenotypically, and likely index the same developmental processes. More genetically‐informed studies of brain organisation are needed to map those major dimensions onto the relevant biological pathways and mechanisms.

We suggest a similar organisation of phenotypic and genetic brain architecture is supporting evidence for the neurobiological validity of canonical brain networks. The fact that our theoretical grouping of volumes into brain networks—informed by commonly‐referenced studies of structural, functional and lesion‐based studies (Bressler & Menon, 2010; Jung & Haier, 2007; Madole et al., 2021; Menon & Uddin, 2010)—yielded networks that explained more variance than expected by randomly grouping volumes into networks, provides some evidence for the ontological reality of those networks.

However, it was surprising to find a lack of specificity between different networks at the level of their broad associated polygenic signal, which was quantified through very high genetic correlations between genetic PC1s underlying brain volumes in different canonical networks (range *r*_*g*_ = 0.63–0.97). This suggests that our Genomic PCA analyses captured general genes linked with global brain‐wide features of morphometric trends, which are practically the same across canonical networks and the whole brain. Future studies wishing to index the genetic correlates of these global features may focus on more parsimonious and computationally more efficient brain networks, including only few volumes most representative of overall brain structure (e.g., 8 regions in central executive network) rather than modelling the whole brain.

### 4.2 Genetic correlations between general cognitive ability and general morphometry underlying canonical brain networks

Using a multivariate definition of general cognitive ability, we demonstrated PC1s underlying all nine brain networks and the whole brain, were genetically associated with cognitive ability at small‐to‐moderate magnitudes (*r*_*g*_ = 0.17–0.21). The effect sizes were about the same magnitude as Jansen et al. (2020) found for a genetic correlation between total brain volume and cognitive ability (*r*_*g*_ = 0.24); this was even when some of our models considered only few brain regions (i.e., central executive included only 8 volumes and still yielded magnitudes as large as total brain size). Furthermore, our cogitive ability ‐ genetic network correlations were numerically larger than genetic correlations obtained from individual brain volumes (range *r*_*g*_ = 0.07–0.13 in Biton et al., 2020). Extracting PC1s seems to distil less noisy genetic variance, which is more robustly relevant to cognitive ability.This should encourage future studies to model general trends of morphometry underlying multiple brain regions, instead of considering individual regions only.

In contrast to phenotypic findings (Madole et al., 2021), there was no evidence that genetic correlates underlying morphometry in the central executive network were any more strongly associated with cognitive ability than the other brain networks. This is compatible with the lack of specificity between different brain networks at the level of their associated polygenic signal (discussed in Section 4.1): each network made a similar prediction of cognitive ability at the genetic level. The fact that a disproportionate role of the central executive network did not replicate in our genetically‐informed design (even when accounting for network size), may suggest that genetics are more likely to predispose towards more general genes of global brain features shared across the brain. Tentatively, this would also suggest that instead of genes, environmental processes might drive phenotypically observed specialisations of brain networks, causing different morphometric structures to matter more (or less) for optimal cognitive performance.

### 4.3 Genetic associations between ageing and general dimensions of brain morphometry

We demonstrated that regions genetically more important to overall brain structure (i.e., large whole‐brain PC1 loadings) also tended to be more sensitive towards age‐related shrinkage (i.e., cross‐sectional volume‐age correlations; *r* = −0.27). This may be due to more strenuous metabolic burden (or other functional stresses) on regions central to the overall structure, possibly through more heavily‐demanding cognitive processes. This could alter disproportionately the speed at which some regions atrophy with advancing age. Whereas this was previously described phenotypically, to our knowledge we present the first genetically‐informed study to show this relation. However, we suggest it requires triangulation either by future longitudinal ageing studies, or cross‐sectional studies modelling within‐person atrophy by incorporating information on prior brain size (e.g., intracranial volume as a proxy for size at younger age).

We also found a substantial genetic correlation of general trends of morphometry across the whole brain with the brain age gap (*r*_*g*_ = −0.34), suggesting there is a shared genetic basis to brain age and general trends of brain organisation, even after residualising brain volume GWAS for age. The genetics associated with younger‐appearing brains may act through overlapping biological processes that also underlie mechanisms of well‐integrated global brain morphometric features. That is, patterns of brain structural ageing may not just capture how quickly an individual's regional volumes decline compared to their peers, but rather general healthy morphometry across the brain. This would be compatible with phenotypic research showing that younger brain age predicts better physical fitness, better fluid intelligence and longer lifespan (Cole et al., 2018). Healthy brain morphometry could vary between people for many non‐age‐related reasons; our findings suggest it may, at least partly, be due to genetic predisposition, possibly towards better‐integrated, more resilient brain biology.

### 4.4 Limitations

Analyses in this study come with limitations. Genetic correlations are representative of genetic associations across the entire genome, but do not give direct insight into specific DNA regions of sharing. As genetic correlations were calculated using LDSC, the limitations that apply to LDSC methodology apply to our study (discussion in Supplementary Note; Appendix S1). We conclude based on heritability estimates, indexing signal‐to‐noise ratios in GWAS, that there was sufficient polygenic signal to warrant LDSC analysis (heritability ranged 7–42%). LDSC intercepts were perfectly associated with phenotypic correlations (*R* ^*2*^ = 0.99), indicating that the analyses successfully separated confounding signals (including environmental factors) from the estimates of genetic correlations.

This study was conducted in the UK Biobank sample, which is not fully representative of the general population of the United Kingdom: its participants are more wealthy, healthy and educated than average (Fry et al., 2017). Cohort effects may affect the degree to which differential brain‐regional susceptibility to ageing can be inferred from cross‐sectional data. It remains to be tested whether our results can be extrapolated to socio‐economically poorer subpopulations, or outside European ancestry. Results were also dependent on the choice of brain parcellation to divide the cortex into separate regions.

### 4.5 Conclusion

To study the neurobiological bases of adult cognitive ageing, we introduced a multivariate framework to integrate covariance across multiple brain regions and the genome (Genomic PCA), which allowed modelling of general dimensions underlying brain‐wide morphometry. In line with Cheverud's Conjecture, phenotypic and genetic brain organisation seemed to operate on the same major dimensions and moderate genetic correlations supported that genes underlying general dimensions of brain morphometry are implicated in cognitive ageing. Genetically more important regions to overall brain structure tended to be more susceptible towards age‐related shrinkage. However, instead of uncovering localised brain network‐specific genetic correlates, we only found evidence for general genetic correlates of brain‐wide morphometric features. This may imply that environmental, or otherwise non‐genetic, processes are more likely than genes to drive different morphometric structures to matter more (or less) for better cognitive performance. The evidence presented here brings us closer to characterising genetic etiology and robust neurobiological correlates of cognitive ageing, and provides a foundation for future investigations ultimately working on interventions for cognitive decline.

## AUTHOR CONTRIBUTIONS

Conceptualisation and methodology: Stuart J. Ritchie, Elliot M. Tucker‐Drob, James H. Cole, Anna E. Fürtjes, Simon R. Cox. Supervision: Stuart J. Ritchie, Elliot M. Tucker‐Drob, James H. Cole. Network characterisation: Simon R. Cox. Idea to investigate genetic brain age gap—genetic PC1 correlation: James W. Madole. Function used to perform genetic parallel analysis: Javier de la Fuente. Data access: Cathryn M. Lewis. Genetic quality control: Anna E. Fürtjes, Jonathan R. I. Coleman. GWAS calculation: Anna E. Fürtjes, Ryan Arathimos. Data analysis: Anna E. Fürtjes. Writing: Anna E. Fürtjes. Visualisations: Anna E. Fürtjes. Reviewed draft: all authors.

## CONFLICT OF INTEREST STATEMENT

Ian Deary is a participant in UK Biobank. All other authors have no conflicts of interest to declare.

## Supporting information


**Appendix S1.** Supplementary Information.

**Figure S1.** Supplementary Figures.

**Table S1.** Supplementary Tables.

## Data Availability

Access to phenotypic and genetic UK Biobank data was granted through the approved application 18177. We have made the 83 GWAS summary statistics of regional volumes available at the GWAS catalogue (https://www.ebi.ac.uk/gwas/). GWAS summary statistics for the seven cognitive traits by (de la Fuente et al., 2021) were downloaded at https://datashare.ed.ac.uk/handle/10283/3756. The pre‐registration for this analysis can be found online (https://osf.io/7n4qj). Full analysis code including results for this study are available at https://annafurtjes.github.io/Genetic_networks_project/index.html.
