# Supplementary material for: General dimensions of human brain morphometry inferred from genome‐wide association data
Source: Hum Brain Mapp. 2023 Mar 29;44(8):3311–23. doi: 10.1002/hbm.26283 (PMC10171533; doi:10.1002/hbm.26283)
Supplement: Supplementary file 1 — Appendix S1. Supplementary Information. [file HBM-44-3311-s003.docx]

# **Supplementary Methods**

### **1. Study Design**

#### 1.1. UK Biobank data

The UKB study received ethical approval by the Research Ethics Committee. All participants signed informed consent for their data to be analysed. Access to phenotypic and genetic UK Biobank data was granted through the approved application 18177. Magnetic resonance imaging **(**MRI) data was collected by the UK Biobank study with identical hardware and software in Manchester, Newcastle, and Reading. Brain volumetric phenotypes were pre-processed by an imaging-pipeline developed and executed on behalf of UK Biobank (Alfaro-Almagro et al., 2018). More information on T1 processing can be found in the UK Biobank online documentation (Smith, Alfaro-Almagro, et al., 2020). Briefly, cortical surfaces were modelled using FreeSurfer, and 33 volumes were extracted based on Desikan-Killiany surface templates (Desikan et al., 2006) (UKB does not provide frontal pole measures due to high missingness); 8 subcortical areas were derived using FreeSurfer aeseg tools (Fischl et al., 2002). Volumetric measures (mm^3^) have been generated in each participant’s native space. We used 83 available imaging-derived phenotypes (IDPs) of cortical and subcortical grey-matter volumes in regions of interest spanning the whole brain (UK Biobank category 192 & 190; STable 1). We assume the IDPs to be normally-distributed.

#### 1.2. Phenotypic quality control

Excluding participants who withdrew consent, we considered 41,776 participants with non-missing T1-weighted IDPs that had been processed in conjunction with T2-weighted FLAIR (UK Biobank field ID 26500) where available. Using both T1 and T2 measures ensures more precise cortical segmentation (Lindroth et al., 2019). Extreme outliers outside of 4 standard deviations from the mean were excluded, which ranged between 41,686 to 41,769 available participants between different IDP. 381 participants were excluded as they self-reported non-European ethnicity. (In GWAS it is necessary to include unrelated, ethnically homogeneous participants. Different ethnicities display different linkage disequilibrium structures across the genome, and the analyses conducted here would be uninterpretable if conducted across ethnicities. To maximise sample size, we analyse European participants, as they represent the largest ethnically homogeneous group in the UKB cohort). Across the 83 brain volumes and the covariates, this phenotypic quality control resulted in 39,947 complete cases, for whom the following genetic quality control steps were performed.

#### 1.3. Genetic quality control

Out of the 39,947 UK Biobank participants, genetic data were available for 38,957 participants. Genetic data was quality controlled by UK Biobank and were downloaded from the full release (Bycroft et al., 2018). We applied additional quality control as previously described in Coleman et al. (2020) using PLINK2 (Chang et al., 2015). 38,038 participants were of European ancestry according to 4-means clustering on the first two genetic principal components available through UK Biobank (Warren et al., 2017). Of those participants, we removed 72 due to quality assurance issues reported by UK Biobank and 204 participants due to high rates of missingness (2% missingness). To obtain a sample of unrelated individuals, 956 participants were removed using the greedyRelated algorithm (KING *r* < 0.044 (Manichaikul et al., 2010)). The algorithm is “greedy” because it maximises sample size; for example, it removes the child in a parent-child-trio. Finally, 28 participants were removed because genetic sex did not align with self-reported sex, resulting in a total of 36,778 participants (STable 10). Genetic sex was identified based on measures of X-chromosome homozygosity (*F*_X_ ; removal of participants with *F*_X_ < 0.9 for phenotypic males, *F*_X_ > 0.5 for phenotypic females). The final sample (*N* = 36,778) included 19,888 females (54 %) and had an average age of 63.3 years at the neuroimaging visit (range from 40.0 to 81.8 years).

Out of 805,426 available directly genotyped variants, 104,771 were removed for high rates of missing genotype data (> 98%). 103,137 variants were removed due to a minimum allele frequency of 0.01, and 9,935 variants were removed as they failed the Hardy-Weinberg exact test (*p*-value = 10^-8^). After excluding 16,326 variants on the sex chromosomes and those with chromosome labels larger than 22, we obtained a final sample of 571,257 directly genotyped SNPs. Imputed genotype data was obtained by UK Biobank with reference to the Haplotype Reference Consortium (McCarthy et al., 2016), and we filtered them for a minor allele frequency of above 0.01 and an IMPUTE INFO metric of above 0.4.

#### 1.4. Measures of cognitive performance

In this study, we considered GWAS summary statistics of performance in seven cognitive tests by de la Fuente et al. (2021) that were calculated with between 11,263 and 331,679 participants for each test. We considered the HapMap 3 reference SNPs with the MHC regions removed. These seven cognitive performance tests were assessed on a touchscreen computer: *Matrix Pattern Completion task* for nonverbal reasoning, *Memory – Pairs Matching Test* for memory, *Reaction Time* for perceptual motor speed, *Symbol Digit Substitution Task* for information processing speed, *Trail Making Test – B* and *Tower Rearranging Task* for executive functioning, and *Verbal Numerical Reasoning Test* for verbal and numeric problem solving, or fluid intelligence. Despite the non-standard and unsupervised delivery of assessment, these cognitive tests demonstrate strong concurrent validity compared with standard reference tests (*r* = .83) and good test-retest reliability (Pearson *r* range for different cognitive tests = 0.4–0.78) (Fawns-Ritchie & Deary, 2020).

### **2. Statistical analysis**

#### 2.1. GWAS summary statistics calculation

The 83 regional brain volumes described in Supplementary Methods 1.1. were quality controlled as described in Supplementary Methods 1.2-1.3. GWAS summary statistics for these 83 regional brain volumes (continuous variables) were calculated using REGENIE (Mbatchou et al., 2021), which fits polygenic effects in a linear mixed model using Ridge regression. The REGENIE pipeline is split into two steps: First, blocks of directly genotyped SNPs are used to fit a cross-validated whole-genome regression model using Ridge regression, to determine the amount of phenotypic variance explained by genetic effects. Second, the association between the phenotype and imputed genetic variants is calculated conditional upon Ridge regression predictions from the first step. Proximal contamination is circumvented by using a leave-one-chromosome-out scheme.

Covariates included in the GWAS analyses were *age at neuroimaging visit*, *sex*, *genotyping* *batch*, and *40 genetic principal components* as provided by UK Biobank. We also derived the variables *time of year*, *head position*, and *acquisition site,* but excluded them from our set of GWAS covariates because they were not associated with the brain volumes at the pre-registered arbitrary cut-off of *r* ≤ .10 (STable 9), and therefore explained less than 1% of the phenotype variance. Note that, in contrast to other existing brain-volume GWAS in UK Biobank (e.g., Smith, Douaud, et al., 2020), our analyses were conducted *without* controlling for brain size (or any other global brain measure such as total grey-matter volume or intracranial volume). This is because we wanted to represented total variance associated with regional volumes, rather than capturing variance that persisted above and beyond variance that mapped onto global measures (also discussed in the introduction of the manuscript). Genetic correlations calculated relative to such global measures are known to attenuate genetic correlations among volumes, as well as with other traits such as cognitive abilities (de Vlaming et al., 2021). In the context of this study, we aim to model general dimensions of variance shared between brain volumes which will probably closely covary with overall brain size. Attenuated genetic correlations would hide major dimensions of variance across genetic brain networks, because much of the variance shared between volumes overlaps with variance indexed by brain size and would therefore not tag general dimensions of shared genetic variance between brain volumes. This variance is of interest because general intelligence yields global rather than a region-specific associations with grey matter volume (Hilger et al., 2020). Equally, aging affects the whole brain rather than individual regions (Cole et al., 2019).

#### 2.2. Genetic and phenotypic correlation matrices between brain volumes.

To derive dimensions of shared morphometry across brain volumes, we calculated both a phenotypic and a genetic correlation matrix from 83 grey-matter volumes. Phenotypic regional brain volumes were residualised for age at neuroimaging visit and sex, and then used to estimate a phenotypic correlation matrix through Pearson’s correlations with complete pairwise observations. The genetic correlation matrix was inferred through LDSC (Bulik-Sullivan et al., 2015), a technique quantifying shared genome-wide polygenic effects between traits using GWAS summary statistics. Cross-trait LDSC regresses the product of effect sizes in two GWAS onto linkage disequilibrium (LD) scores, indicating how correlated a genetic variant is with its neighbouring variants (Bulik-Sullivan et al., 2015). The slope indexes the genetic correlation, while the intercept captures signal uncorrelated with LD, such as population stratification, environmental confounding, and sample overlap (Lee et al., 2018). Interregional genetic correlations ranged from *r_g_* = -0.08 (*SE* = 0.013) between right frontal pole and left pallidum, to *r_g_* = 0.87 (*SE* = 0.017) between left middle temporal and left inferior temporal (*Fig.3B, SFig.1*). Corresponding standard errors ranged between 0.01 and 0.03 (mean = 0.014; *SD* = 0.002).

#### 2.3. Comparing phenotypic and genetic correlations

To quantify the relationship between phenotypic and genetic correlations, we estimated the correlation between 3403 phenotypic and 3403 genetic interregional brain volume correlations ($\frac{83(83-1)}{2}$ = 3403 correlations between 83 volumes). We calculated genetic correlation matrices indicating genetic overlap between the 83 volumes using linkage disequilibrium score regression (LDSC; Bulik-Sullivan et al., 2015) as implemented in the GenomicSEM software (Grotzinger et al., 2019; Fig.2.2). Genetic between-volume correlations are displayed in *SFig.1-10*. To obtain comparable indices of phenotypic covariance, we ran PCA on a phenotypic correlation matrix obtained from the same brain volume variables used to calculate GWAS.

#### 2.4. Principal component analysis (PCA) of genetic and phenotypic correlation matrices

PCA was applied to the phenotypic and genetic correlation matrices indicating genetic overlap between brain volumes described above to obtain their respective first principal component (PC). The first PC1 represents an underlying dimension of common structural sharing across regional volumes, which we refer to as general dimensions of shared morphometry throughout this manuscript. PC1 loadings were calculated for all volumes in the whole brain, as well as theoretical grouping of fewer volumes thought to reside in smaller canonical networks to quantify contributions of regional volumes to this either brain-wide, or network-specific dimension of shared morphometry. Phenotypically, this was done using the eigen function in R, and genetically this was done following the pipeline outlined in detail in the main Methods section (*Fig.2*).

#### 2.5. Genome-wide PCs of morphometry across the whole brain and canonical networks

To statistically represent volumetric PCs on a genome-wide level, we averaged genome-wide SNP effects contributed by multiple grey-matter volume GWAS summary statistics, weighted by their respective (region-specific) PC1 loadings. We obtained one set of GWAS summary statistics showing SNP associations of a genetic principal component underlying multiple GWAS phenotypes derived from samples of unknown degrees of overlap. SNP effects were calculated by adapting existing software for genome-wide multivariate meta-analysis by Baselmans et al. (2019). More details are in the main Methods section (*Fig.2*).

We tested and validated this novel approach in an independent set of GWAS summary statistics of four risky behaviours (Linnér et al., 2019). In addition to the risky behaviour GWAS, another set of summary statistics is available for a phenotypic PC1 underlying these risky behaviour phenotypes that the authors had calculated phenotypically before running GWAS analyses. We compared these phenotypic PC1 GWAS summary statistics provided by Linnér et al. (2019) with summary statistics for a *genetic* PC1 underlying the four risky behaviours GWAS that we calculated using Genomic PCA (*Fig.2*). They were genetically correlated at *r_g_* = 0.99 (*SE* = 0.037) confirming that our method captures the same signal as can be obtained from phenotypic PCs, by simply relying on publicly available GWAS data. For details of the analysis and code refer to: <https://annafurtjes.github.io/genomicPCA/> .

#### 2.6. Parallel analysis

We tested whether genetic PCs underlying multiple volumes in the whole brain, and canonical brain networks explained more variance than expected by chance, that is, whether they explained more than 95% of their corresponding PCs generated under a simulated null correlation matrix. We developed a version of parallel analysis to generate null distributions of eigenvalues by simulating null correlation matrices sampled from a diagonal population correlation matrix, where the multivariate sampling distribution is specified to take the form of the sampling distribution of the standardised empirical genetic correlation matrix (the V_STD_ matrix, as estimated using GenomicSEM (Grotzinger et al., 2019)). This sampling correlation matrix serves as an index of the precision of the elements in the empirical genetic covariance matrix (i.e., heritabilities and co-heritabilities across traits) and the sampling dependencies among these when generating the random null models. We specified 1,000 replications to simulate the null correlation matrices and used a 95% threshold for distinguishing true eigenvalues from noise.

#### 2.7. Simulation of networks with randomly included brain volumes.

We performed an additional sensitivity analysis simulating networks with randomly included brain volumes, to determine whether shared structural variance relied on network membership, or arose through phenotypic properties common to all regional brain volumes. To compare explained variances between canonical networks and random networks, we quantified the expected explained variance in random networks by randomly sampling regions 800 times each, for different numbers of included volumes (because networks including fewer volumes generally tend to explain a larger percentage of variance, as larger networks are more heterogeneous). That is, simulations were run for 8, 10, 12, 16, 30, and 36 included regions, to obtain a distribution for each networks size to compare the corresponding network’s explained variance to. We reported the mean explained variance by PCs for networks with randomly included volumes and a 95% confidence interval. Comparisons between explained variances for random and empirical networks were done for the same number of included volumes.

#### 2.8. Comparing phenotypic and genetic loadings onto PC1 underlying 83 brain-wide volumes

To compare whether genetic correlation structures of regional brain morphometry resembled the phenotypic correlation structure of the same regions, we calculated an un-standardised linear regression with a vector of 83 phenotypic whole-brain PC1 loadings as the dependent variable, and a vector containing 83 genetic whole-brain PC1 loadings as the independent variable. We calculated the Tucker congruence coefficient to quantify the relative similarity between the two sets of PC1 loadings independent of their absolute magnitude. The coefficient is insensitive to scalar multiplication (Tucker, 1951).

#### 2.9. Correlation between genetic loadings onto PC1 underlying 83 brain-wide volumes with age sensitivity

Pearson’s correlations between 83 phenotypic grey-matter volumes and age at neuroimaging visit were calculated to quantify cross-sectional age-volume-correlations for each of the 83 brain volumes. These age-volume correlations are referred to as *age sensitivity* throughout the manuscript. We estimated the correlation between a vector containing indices of age sensitivity and (1) a vector of *genetic* whole-brain PC1 loadings, and for comparison (2) a vector of *phenotypic* whole-brain PC1 loadings.

#### 2.10. Genetic correlation between general dimensions of shared morphometry across the whole-brain and brain age

Using cross-trait LDSC (Bulik-Sullivan et al., 2015), we calculated a genetic correlation between genetic PC1 underlying brain-wide volumes (as created by Genomic PCA) and the *brain age* gap. We used the brain age GWAS summary statistics by Kaufmann et al. (2019). Brain age is a phenotype based on individual-level predictions of how much older (or younger) an individual’s brain appears, relative to their chronological age. It is estimated using parameters characterising the relationship between age and structural neuroimaging measures (volume, thickness, and surface area) that were tuned using machine learning in an independent sample. The final brain age phenotype indexed in the GWAS was calculated as the difference between participants chronological age and their age as predicted based on structural brain characteristics.

#### 2.11. Genomic SEMs of genetic correlations between structural brain networks and a factor of general cognitive ability

We assessed genetic correlations between genetic PC1s underlying canonical brain networks and general cognitive ability using GenomicSEM (Grotzinger et al., 2019) (*Fig.5*). In GenomicSEM (Grotzinger et al., 2019), the genetic general cognitive ability factor was modelled from seven cognitive ability GWAS summary statistics (described in Supplementary Methods 1.4.), and the genetic correlation between genetic general cognitive ability and genetic brain PCs was estimated with diagonally weighted least squares. To quantify model fit, we reported default fit indices calculated by the GenomicSEM package: *χ2* values, the Akaike Information Criterion (AIC), the Comparative Fit Index (CFI) and the Standardised Root Mean Square Residuals (SRMR). The multiple testing burden was addressed by correcting *p*-values from the genetic correlations for multiple testing with a false-positive discovery rate of 5% (Benjamini & Hochberg, 1995).

We preregistered that we would test for significant differences in correlation magnitudes between the networks whose underlying PC1 yielded a significant association with general cognitive abilities. Because we hypothesised a particularly strong association for the central executive network, we planned to perform this comparison between the central executive and all other networks, to reduce the multiple testing burden. We fitted two GenomicSEM models in which correlation magnitudes between general cognitive ability and both the central executive and another network were either freely estimated, or they were forced to be the same. A significant decrease in model fit (as indicated as the difference in *χ2*) between the freely estimated model and the constrained model (*df* = 1) would indicate that there likely are differences in correlation magnitudes between the networks in how strongly they correlate with general cognitive ability (*SFig.22*). We found no evidence that the PC1 underlying the central executive network was any more genetically correlated with cognitive ability than the PC1 underlying any other network (STable 5).

Additionally, we assessed whether the PC1 underlying the central executive network was disproportionately genetically correlated with general cognitive ability considering its small size (i.e., few included volumes). Similar to the approach described above, we fitted two models: One, in which we freely estimate the correlation between the genetic PC1 underlying the central executive and general cognitive ability, and the correlation between genetic PC1 underlying another network and general cognitive ability. We then divided the correlation magnitude by the number of regions included in the network (i.e., magnitude was divided by 8 for the central executive network, it was divided by 16 for the default mode, by 36 for the P-FIT etc.). The second model had the same set up, but we forced the adjusted correlations of the two networks with cognitive ability to be equal (e.g., r_central executive_ / 8 == r_default_ / 16). We assessed whether there was a significant difference in ***χ^2^*** model fit between these two models. As above, a significant decrease in model fit between the freely estimated model and the constrained model (*df*  = 1) would indicate that there likely are differences in relative correlation magnitudes (i.e., magnitudes adjusted for network sizes). Based on previous findings, we expected the relative magnitude for the central executive network to be significantly larger than the relative magnitude for any other network. Even when accounting for network size, we found no evidence that the genetic PC1 underlying the central executive network was any more genetically correlated with cognitive ability than a genetic PC1 underlying any other canonical network (STable 6).

To probe whether any specific cognitive ability might have driven the genetic correlation between PCs underlying brain networks and general cognitive ability, we reported genetic correlations between the significant networks and three specific cognitive abilities: (1) *Matrix Pattern Completion task* to represent nonverbal reasoning, (2) *Memory – Pairs Matching Test* to represent memory, and (3) *Symbol Digit Substitution Task* to represent information processing speed. Reducing the analyses to only three consistent and representative cognitive measures reduced the burden of multiple testing. Matrix Pattern Completion consistently yielded the strongest genetic correlations with PCs underlying all the brain networks (mean *r_g_* across different networks = 0.18). Genetic correlations for Symbol Digit Substitution Task were slightly smaller (mean *r_g_* = 0.12), followed by Memory which had the lowest average correlations (mean *r_g_* = 0.09; STable 7).

We also calculated *Q_trait_* heterogeneity indices (Grotzinger et al., 2022) to evaluate whether the general cognitive ability factor that we fit in the models above accounts well for the specific cognitive abilities. To this end, we compared the fit of two models for each network as displayed in *SFig.23*. One model allows for independent associations between the seven cognitive traits, and both general cognitive ability and the PC1 underlying the considered brain network. The second model forces the association between the seven cognitive traits and the PC1 underlying the brain network to go through the general cognitive ability factor. We obtained ***χ^2^*** fit statistics for both models and tested their difference for statistical significance (**∆ *χ^2^*** ≠ 0; *df* = 6). Non-significant results (*p* > 0.05/10) would suggest that genetic associations between cognitive abilities and brain networks are likely general and act through a factor of general cognitive ability. We obtained non-significant *Q_trait_* heterogeneity indices (Grotzinger et al., 2022) for all brain networks, demonstrating that the general cognitive ability factor accounted well for the patterns of association between specific cognitive abilities and the brain networks (*SFig.23*).

**Data and code availability.** Access to phenotypic and genetic UK Biobank data was granted through the approved application 18177. We have made the 83 GWAS summary statistics of regional volumes available at the GWAS catalogue (<https://www.ebi.ac.uk/gwas/>). GWAS summary statistics for the seven cognitive traits by (de la Fuente et al., 2021) were downloaded at <https://datashare.ed.ac.uk/handle/10283/3756>. The pre-registration for this analysis can be found online (<https://osf.io/7n4qj>). Full analysis code including results for this study are available at <https://annafurtjes.github.io/Genetic_networks_project/index.html>.

References

Alfaro-Almagro, F., Jenkinson, M., Bangerter, N. K., Andersson, J. L. R., Griffanti, L., Douaud, G., . . . Smith, S. M. (2018). Image Processing and Quality Control for the First 10,000 Brain Imaging Datasets from Uk Biobank. *NeuroImage, 166*, 400-424. doi:10.1016/j.neuroimage.2017.10.034

Baselmans, B. M. L., Jansen, R., Ip, H. F., van Dongen, J., Abdellaoui, A., van de Weijer, M. P., . . . Social Science Genetic Association, C. (2019). Multivariate Genome-Wide Analyses of the Well-Being Spectrum. *Nature Genetics, 51*(3), 445-451. doi:10.1038/s41588-018-0320-8

Benjamini, Y., & Hochberg, Y. (1995). Controlling the False Discovery Rate: A Practical and Powerful Approach to Multiple Testing. *Journal of the Royal Statistical Society: Series B (Methodological), 57*(1), 289-300. doi:10.1111/j.2517-6161.1995.tb02031.x

Bulik-Sullivan, B., Finucane, H. K., Anttila, V., Gusev, A., Day, F. R., Loh, P.-R., . . . Robinson, E. B. (2015). An Atlas of Genetic Correlations across Human Diseases and Traits. *Nature Genetics, 47*(11), 1236. doi:10.1038/ng.3406

Bycroft, C., Freeman, C., Petkova, D., Band, G., Elliott, L. T., Sharp, K., . . . Marchini, J. (2018). The Uk Biobank Resource with Deep Phenotyping and Genomic Data. *Nature, 562*(7726), 203-209. doi:10.1038/s41586-018-0579-z

Chang, C. C., Chow, C. C., Tellier, L. C., Vattikuti, S., Purcell, S. M., & Lee, J. J. (2015). Second-Generation Plink: Rising to the Challenge of Larger and Richer Datasets. *GigaScience, 4*(1). doi:10.1186/s13742-015-0047-8

Cole, J. H., Marioni, R. E., Harris, S. E., & Deary, I. J. (2019). Brain Age and Other Bodily ‘Ages’: Implications for Neuropsychiatry. *Molecular Psychiatry, 24*(2), 266-281. doi:10.1038/s41380-018-0098-1

Coleman, J. R. I., Peyrot, W. J., Purves, K. L., Davis, K. A. S., Rayner, C., Choi, S. W., . . . on the behalf of Major Depressive Disorder Working Group of the Psychiatric Genomics, C. (2020). Genome-Wide Gene-Environment Analyses of Major Depressive Disorder and Reported Lifetime Traumatic Experiences in Uk Biobank. *Molecular Psychiatry, 25*(7), 1430-1446. doi:10.1038/s41380-019-0546-6

de la Fuente, J., Davies, G., Grotzinger, A. D., Tucker-Drob, E. M., & Deary, I. J. (2021). A General Dimension of Genetic Sharing across Diverse Cognitive Traits Inferred from Molecular Data. *Nature Human Behaviour, 5*(1), 49-58. doi:10.1038/s41562-020-00936-2

de Vlaming, R., Slob, E. A. W., Jansen, P. R., Dagher, A., Koellinger, P. D., Groenen, P. J. F., & Rietveld, C. A. (2021). Multivariate Analysis Reveals Shared Genetic Architecture of Brain Morphology and Human Behavior. *Communications Biology, 4*(1), 1180. doi:10.1038/s42003-021-02712-y

Desikan, R. S., Ségonne, F., Fischl, B., Quinn, B. T., Dickerson, B. C., Blacker, D., . . . Hyman, B. T. (2006). An Automated Labeling System for Subdividing the Human Cerebral Cortex on Mri Scans into Gyral Based Regions of Interest. *NeuroImage, 31*(3), 968-980. doi:10.1016/j.neuroimage.2006.01.021

Fawns-Ritchie, C., & Deary, I. J. (2020). Reliability and Validity of the Uk Biobank Cognitive Tests. *PLOS ONE, 15*(4), e0231627. doi:10.1371/journal.pone.0231627

Fischl, B., Salat, D. H., Busa, E., Albert, M., Dieterich, M., Haselgrove, C., . . . Dale, A. M. (2002). Whole Brain Segmentation: Automated Labeling of Neuroanatomical Structures in the Human Brain. *Neuron, 33*(3), 341-355. doi:10.1016/S0896-6273(02)00569-X

Grotzinger, A. D., Mallard, T. T., Akingbuwa, W. A., Ip, H. F., Adams, M. J., Lewis, C. M., . . . Nivard, M. G. (2022). Genetic Architecture of 11 Major Psychiatric Disorders at Biobehavioral, Functional Genomic and Molecular Genetic Levels of Analysis. *Nature Genetics, 54*, 548–559. doi:10.1038/s41588-022-01057-4

Grotzinger, A. D., Rhemtulla, M., de Vlaming, R., Ritchie, S. J., Mallard, T. T., Hill, W. D., . . . Tucker-Drob, E. M. (2019). Genomic Structural Equation Modelling Provides Insights into the Multivariate Genetic Architecture of Complex Traits. *Nature Human Behaviour, 3*(5), 513-525. doi:10.1038/s41562-019-0566-x

Hilger, K., Winter, N. R., Leenings, R., Sassenhagen, J., Hahn, T., Basten, U., & Fiebach, C. J. (2020). Predicting Intelligence from Brain Gray Matter Volume. *Brain Structure and Function, 225*(7), 2111-2129. doi:10.1007/s00429-020-02113-7

Kaufmann, T., van der Meer, D., Doan, N. T., Schwarz, E., Lund, M. J., Agartz, I., . . . Karolinska Schizophrenia, P. (2019). Common Brain Disorders Are Associated with Heritable Patterns of Apparent Aging of the Brain. *Nature Neuroscience, 22*(10), 1617-1623. doi:10.1038/s41593-019-0471-7

Lee, J. J., McGue, M., Iacono, W. G., & Chow, C. C. (2018). The Accuracy of Ld Score Regression as an Estimator of Confounding and Genetic Correlations in Genome-Wide Association Studies. *Genetic Epidemiology, 42*(8), 783-795. doi:10.1002/gepi.22161

Lindroth, H., Nair, V. A., Stanfield, C., Casey, C., Mohanty, R., Wayer, D., . . . Sanders, R. D. (2019). Examining the Identification of Age-Related Atrophy between T1 and T1 + T2-Flair Cortical Thickness Measurements. *Scientific Reports, 9*(1), 11288. doi:10.1038/s41598-019-47294-2

Linnér, R. K., Biroli, P., Kong, E., Meddens, S. F. W., Wedow, R., Fontana, M. A., . . . Social Science Genetic Association, C. (2019). Genome-Wide Association Analyses of Risk Tolerance and Risky Behaviors in over 1 Million Individuals Identify Hundreds of Loci and Shared Genetic Influences. *Nature Genetics, 51*(2), 245-257. doi:10.1038/s41588-018-0309-3

Manichaikul, A., Mychaleckyj, J. C., Rich, S. S., Daly, K., Sale, M., & Chen, W.-M. (2010). Robust Relationship Inference in Genome-Wide Association Studies. *Bioinformatics, 26*(22), 2867-2873. doi:10.1093/bioinformatics/btq559

Mbatchou, J., Barnard, L., Backman, J., Marcketta, A., Kosmicki, J. A., Ziyatdinov, A., . . . Marchini, J. (2021). Computationally Efficient Whole-Genome Regression for Quantitative and Binary Traits. *Nature Genetics, 53*, 1097–1103. doi:10.1038/s41588-021-00870-7

McCarthy, S., Das, S., Kretzschmar, W., Delaneau, O., Wood, A. R., Teumer, A., . . . the Haplotype Reference, C. (2016). A Reference Panel of 64,976 Haplotypes for Genotype Imputation. *Nature Genetics, 48*(10), 1279-1283. doi:10.1038/ng.3643

Smith, S., Alfaro-Almagro, F., & Miller, K. (2020). Uk Biobank Brain Imaging Documentation. Retrieved from <https://biobank.ctsu.ox.ac.uk/crystal/crystal/docs/brain_mri.pdf>

Smith, S. M., Douaud, G., Chen, W., Hanayik, T., Alfaro-Almagro, F., Sharp, K., & Elliott, L. T. (2020). Enhanced Brain Imaging Genetics in Uk Biobank. *bioRxiv*, 2020.2007.2027.223545. doi:10.1101/2020.07.27.223545

Tucker, L. R. (1951). *A Method for Synthesis of Factor Analysis Studies*. Retrieved from

Warren, H. R., Evangelou, E., Cabrera, C. P., Gao, H., Ren, M., Mifsud, B., . . . Understanding Society Scientific, g. (2017). Genome-Wide Association Analysis Identifies Novel Blood Pressure Loci and Offers Biological Insights into Cardiovascular Risk. *Nature Genetics, 49*(3), 403-415. doi:10.1038/ng.3768
